# Educational attainment, health outcomes and mortality: a within-sibship Mendelian randomization study

**DOI:** 10.1093/ije/dyad079

**Published:** 2023-06-09

**Authors:** Laurence J Howe, Humaira Rasheed, Paul R Jones, Dorret I Boomsma, David M Evans, Alexandros Giannelis, Caroline Hayward, John L Hopper, Amanda Hughes, Hannu Lahtinen, Shuai Li, Penelope A Lind, Nicholas G Martin, Pekka Martikainen, Sarah E Medland, Tim T Morris, Michel G Nivard, Jean-Baptiste Pingault, Karri Silventoinen, Jennifer A Smith, Emily A Willoughby, James F Wilson, Within Family Consortium, Rafael Ahlskog, Ole A Andreassen, Helga Ask, Archie Campbell, Rosa Cheesman, Yoonsu Cho, Kaare Christensen, Elizabeth C Corfield, Christina C Dahm, Alexandra Havdahl, William D Hill, Shona M Kerr, Antti Latvala, Marianne Nygaard, Teemu Palviainen, Nancy L Pedersen, Robert Plomin, Melissa C Southey, Camilla Stoltenberg, Bjørn Olav Åsvold, Øyvind E Næss, George Davey Smith, Jaakko Kaprio, Ben Brumpton, Neil M Davies

**Affiliations:** Medical Research Council Integrative Epidemiology Unit, Population Health Sciences, University of Bristol, UK; Population Health Sciences, Bristol Medical School, University of Bristol, Bristol, UK; Medical Research Council Integrative Epidemiology Unit, Population Health Sciences, University of Bristol, UK; K.G. Jebsen Center for Genetic Epidemiology, Department of Public Health and Nursing, NTNU, Norwegian University of Science and Technology, Trondheim, Norway; Department of Medicine and Laboratory Sciences, University of Oslo, Oslo, Norway; Department of Community Medicine and Global Health, Institute of Health and Society, Faculty of Medicine, University of Oslo, Oslo, Norway; Department of Biological Psychology, Netherlands Twin Registry, Vrije Universiteit, Amsterdam, Netherlands; Amsterdam Public Health (APH) and Amsterdam Reproduction and Development (AR&D); Medical Research Council Integrative Epidemiology Unit, Population Health Sciences, University of Bristol, UK; University of Queensland Diamantina Institute, University of Queensland, Brisbane, Australia; Institute for Molecular Bioscience, University of Queensland, Brisbane, Australia; Department of Psychology, University of Minnesota, Minneapolis, MN, USA; MRC Human Genetics Unit, Institute of Genetics and Cancer, University of Edinburgh, Western General Hospital, Edinburgh, UK; Centre for Epidemiology and Biostatistics, Melbourne School of Population and Global Health, The University of Melbourne, Parkville, Victoria, Australia; Medical Research Council Integrative Epidemiology Unit, Population Health Sciences, University of Bristol, UK; Population Health Sciences, Bristol Medical School, University of Bristol, Bristol, UK; Population Research Unit, University of Helsinki, Helsinki, Finland; Centre for Epidemiology and Biostatistics, Melbourne School of Population and Global Health, The University of Melbourne, Parkville, Victoria, Australia; Centre for Cancer Genetic Epidemiology, Department of Public Health and Primary Care, University of Cambridge, Cambridge, UK; Precision Medicine, School of Clinical Sciences at Monash Health, Monash University, Clayton, Victoria, Australia; Psychiatric Genetics, QIMR Berghofer Medical Research Institute, Brisbane, Australia; School of Biomedical Sciences, Queensland University of Technology, Brisbane, Australia; Faculty of Medicine, University of Queensland, Brisbane, Australia; Department of Genetics and Computational Biology, QIMR Berghofer Medical Research Institute, Brisbane, Queensland, Australia; Population Research Unit, University of Helsinki, Helsinki, Finland; The Max Planck Institute for Demographic Research, Germany; Department of Public Health Sciences, Stockholm University, Sweden; Psychiatric Genetics, QIMR Berghofer Medical Research Institute, Brisbane, Australia; Faculty of Medicine, University of Queensland, Brisbane, Australia; School of Psychology, University of Queensland, Brisbane, Australia; Medical Research Council Integrative Epidemiology Unit, Population Health Sciences, University of Bristol, UK; Population Health Sciences, Bristol Medical School, University of Bristol, Bristol, UK; Department of Biological Psychology, Netherlands Twin Registry, Vrije Universiteit, Amsterdam, Netherlands; Social Genetic & Developmental Psychiatry Centre, Institute of Psychiatry, Psychology & Neuroscience, King’s College London, London, UK; Department of Clinical, Educational and Health Psychology, University College London, London, UK; Population Research Unit, University of Helsinki, Helsinki, Finland; Department of Epidemiology, School of Public Health, University of Michigan, Ann Arbor, MI, USA; Survey Research Center, Institute for Social Research, University of Michigan, Ann Arbor, MI, USA; Department of Psychology, University of Minnesota, Minneapolis, MN, USA; MRC Human Genetics Unit, Institute of Genetics and Cancer, University of Edinburgh, Western General Hospital, Edinburgh, UK; Centre for Global Health Research, Usher Institute, University of Edinburgh, Teviot Place, Edinburgh, UK; K.G. Jebsen Center for Genetic Epidemiology, Department of Public Health and Nursing, NTNU, Norwegian University of Science and Technology, Trondheim, Norway; Department of Endocrinology, Clinic of Medicine, St Olavs Hospital, Trondheim University Hospital, Trondheim, Norway; Department of Community Medicine and Global Health, Institute of Health and Society, Faculty of Medicine, University of Oslo, Oslo, Norway; Norwegian Institute of Public Health, Oslo, Norway; Medical Research Council Integrative Epidemiology Unit, Population Health Sciences, University of Bristol, UK; Population Health Sciences, Bristol Medical School, University of Bristol, Bristol, UK; Institute for Molecular Medicine FIMM, University of Helsinki, Helsinki, Finland; Medical Research Council Integrative Epidemiology Unit, Population Health Sciences, University of Bristol, UK; K.G. Jebsen Center for Genetic Epidemiology, Department of Public Health and Nursing, NTNU, Norwegian University of Science and Technology, Trondheim, Norway; Medical Research Council Integrative Epidemiology Unit, Population Health Sciences, University of Bristol, UK; K.G. Jebsen Center for Genetic Epidemiology, Department of Public Health and Nursing, NTNU, Norwegian University of Science and Technology, Trondheim, Norway; Division of Psychiatry, University College London, London, UK; Department of Statistical Sciences, University College London, London, UK

**Keywords:** Within-sibship, Mendelian randomization, educational attainment, mortality

## Abstract

**Background:**

Previous Mendelian randomization (MR) studies using population samples (population MR) have provided evidence for beneficial effects of educational attainment on health outcomes in adulthood. However, estimates from these studies may have been susceptible to bias from population stratification, assortative mating and indirect genetic effects due to unadjusted parental genotypes. MR using genetic association estimates derived from within-sibship models (within-sibship MR) can avoid these potential biases because genetic differences between siblings are due to random segregation at meiosis.

**Methods:**

Applying both population and within-sibship MR, we estimated the effects of genetic liability to educational attainment on body mass index (BMI), cigarette smoking, systolic blood pressure (SBP) and all-cause mortality. MR analyses used individual-level data on 72 932 siblings from UK Biobank and the Norwegian HUNT study, and summary-level data from a within-sibship Genome-wide Association Study including >140 000 individuals.

**Results:**

Both population and within-sibship MR estimates provided evidence that educational attainment decreased BMI, cigarette smoking and SBP. Genetic variant–outcome associations attenuated in the within-sibship model, but genetic variant–educational attainment associations also attenuated to a similar extent. Thus, within-sibship and population MR estimates were largely consistent. The within-sibship MR estimate of education on mortality was imprecise but consistent with a putative effect.

**Conclusions:**

These results provide evidence of beneficial individual-level effects of education (or liability to education) on adulthood health, independently of potential demographic and family-level confounders.

Key Messages

- Within-sibship Mendelian randomization can reduce bias from demographic and familial factors that may particularly impact analyses of social and behavioural phenotypes.
- Within-sibship Mendelian randomization indicated that higher educational attainment decreased body mass index, smoking behaviour and blood pressure.
- These findings are consistent with beneficial individual-level health effects of higher (liability to) educational attainment.

## Introduction

Higher educational attainment is strongly associated with better adulthood health and reduced mortality.^1^^,^^2^ However, whether these associations are causal remains unclear because of inconclusive evidence from previous quasi-experimental designs such as the raising of the school leaving age and co-twin control or discordant-twin studies.^3–11^ Another source of evidence on effects of educational attainment on health are Mendelian randomization (MR) studies,^12^ which have used genetic variants associated with educational attainment as instrumental variables to provide consistent evidence for beneficial effects of educational attainment on adulthood health outcomes.^13–17^ A caveat is that educational attainment as measured by years in full-time education is a categorical exposure and so it may be more appropriate to interpret effects in terms of liability to educational attainment.^16^

**Figure 1:**
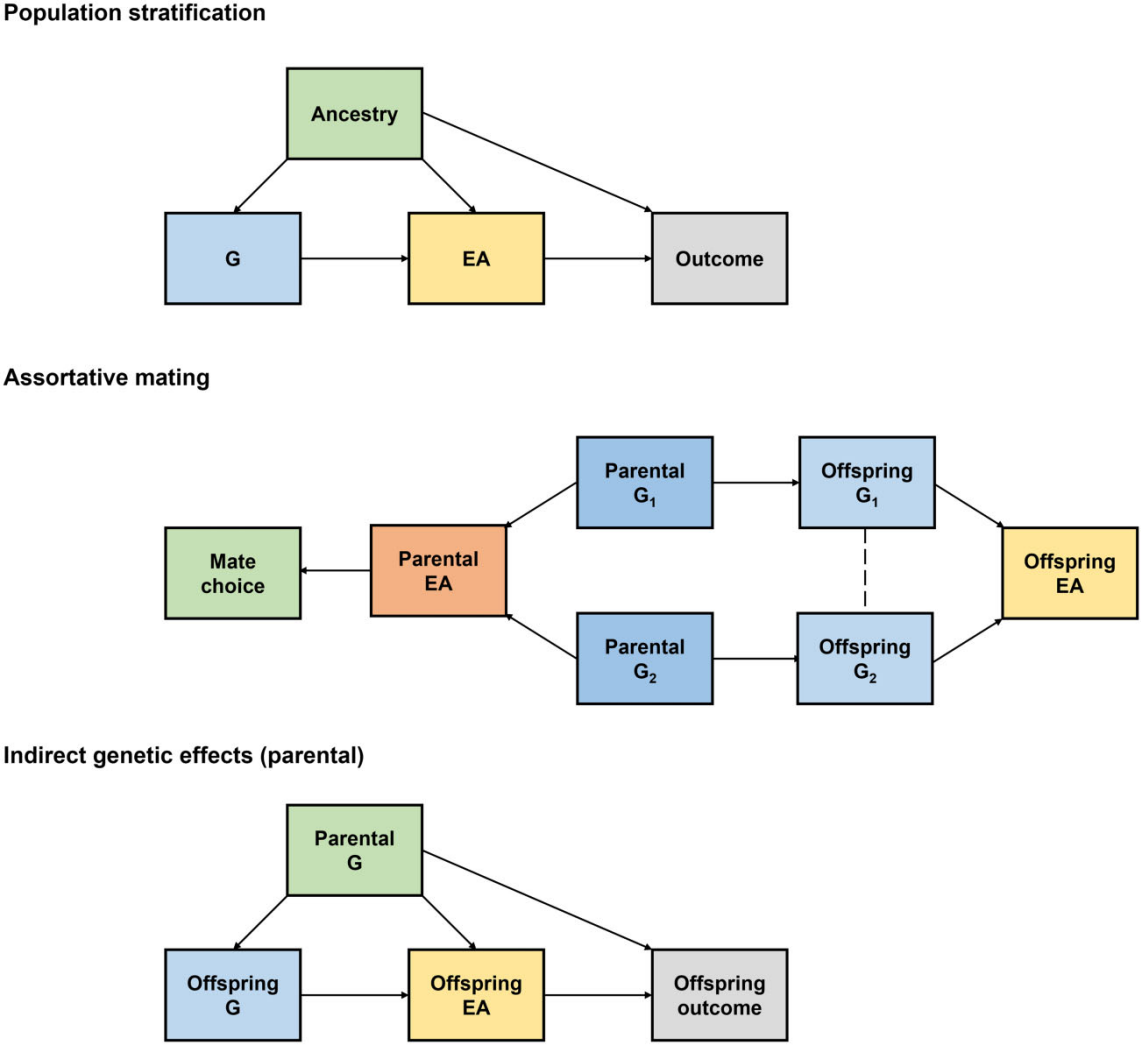
Population stratification, assortative mating and indirect genetic effects. ‘Population stratification’ occurs when ancestry is associated with the allele frequency of the genetic variant (G) and the phenotype of interest, distorting the association between G and the phenotype. In the context of MR, population stratification could distort the association between G and educational attainment (EA) and/or the association between G and the outcome, both of which could lead to bias in MR. ‘Assortative mating’ occurs when a heritable phenotype influences mate choice, e.g. if individuals are more likely to select a partner with a similar EA. Assortative mating leads to correlations for parental genotypes related to assorted phenotypes, which in turn leads to correlations between otherwise independent genotypes in the offspring. For example, if two genetic variants G_1_ and G_2_ influence EA then assortative mating on EA will lead to correlations in offspring for the EA-increasing alleles of G_1_ and G_2_ even if the two alleles are unlinked (linkage disequilibrium = 0). ‘Indirect genetic effects’ occur when the genotypes of relatives (e.g. parents, siblings) influence the phenotypes of the index individual. For example, parents with a higher EA polygenic score may produce an environment for their offspring that is more conducive to learning than parents with a lower EA polygenic score. This has been previously illustrated by evidence that non-transmitted parental EA polygenic scores also associate with offspring phenotypes

A key assumption of MR analyses is that the genetic variant–exposure (here: educational attainment) and genetic variant–outcome (here: health outcomes) associations represent downstream effects of inheriting the genetic variant (or a correlated variant).^12^^,^^18–20^ However, there is growing evidence that genotype–phenotype associations derived from samples of unrelated individuals can reflect other sources of variation^19–21^ (Figure 1). Previous studies have illustrated that Genome-wide Association Study (GWAS) estimates for educational attainment from unrelated individuals reflect population stratification,^22^ assortative mating^18^^,^^23–25^ and indirect genetic effects.^18^^,^^26–29^ Educational attainment is particularly distinctive amongst complex traits because of the large magnitude of indirect genetic effects, the high degree of assortative mating, strong correlations with geographical features and widespread genetic correlations with many phenotypes including health outcomes.^30^ It follows that MR analyses of educational attainment may be biased if using genetic association estimates from unrelated individuals.

Genetic association estimates from within-sibship models are largely robust against population stratification, assortative mating and indirect genetic effects because genetic differences between full siblings are due to random segregation at meiosis.^12^^,^^18–20^^,^^31^ Within-sibship MR using individual-level sibling or within-sibship GWAS data^18^ can control for these sources of genetic association and so can be used to derive less-biased MR estimates.

Here, using individual-level data from UK Biobank and the Trøndelag Health Study, Norway (HUNT) and summary data from a recent within-sibship GWAS,^18^ we generate population and within-sibship MR estimates of the effects of liability to educational attainment on health outcomes and mortality. For health outcomes, we included body mass index (BMI), pack years of cigarette smoking and systolic blood pressure (SBP), which were investigated in previous MR studies and are measured in the majority of UK Biobank and HUNT study participants. We also performed phenotypic analyses using self-reported educational attainment including a twin-based analysis using Finnish twin cohort data.

## Methods

### UK Biobank

UK Biobank is a large-scale prospective cohort study that has been described in detail previously.^32^^,^^33^ In total, 503 325 individuals aged between 38 and 73 years were recruited between 2006 and 2010 from across the UK and attended an assessment centre where they were interviewed, completed a touch-screen questionnaire, and provided various measurements (e.g. height) and biological samples (e.g. blood).

The UK Biobank study sample incidentally includes many related individuals. In our analyses we included individuals with one or more full siblings in the study sample. Siblings were identified in a previous study using the UK Biobank-derived estimates of pairwise identical by state kinships and the proportion of unshared loci (IBS0).^19^^,^^34^ After restricting the sample to sibships with two or more individuals with educational attainment data, our analysis sample included 40 734 individuals from 19 773 sibships. Further detail on the derivation of UK Biobank siblings is contained in the Supplementary methods (available as Supplementary data at *IJE* online).

Educational attainment was defined as in a previous study^35^ using the self-reported qualifications from questionnaire data (field ID: 6138–0.0) to estimate the number of years each individual spent in full-time education. For example, ‘College or University degree’ was mapped to 17 years whereas ‘A levels/AS levels or equivalent’ was mapped to 14 years. Where individuals reported multiple qualifications, the highest qualification in terms of years in education was used. Information on health outcome phenotypes (BMI, smoking, SBP, mortality) and genotyping for UK Biobank study participants is contained in the Supplementary methods (available as Supplementary data at *IJE* online).

### HUNT

HUNT is a series of general health surveys of the adult population of the Trøndelag region, Norway, as detailed in previous publications.^36–38^ Every 10 years, the adult population of this region (∼90 000 adults at the start of HUNT2 in 1995) is invited to attend a health survey (including comprehensive questionnaires, an interview, clinical examination and detailed phenotypic measurements). To date, four health surveys have been conducted, namely HUNT1 (1984–86), HUNT2 (1995–97), HUNT3 (2006–08) and HUNT4 (2017–19), and all surveys have had a >50% participation rate.^39^ In this study, we used data from 32 198 individuals from 12 578 sibships who reported their educational attainment in the HUNT2 survey. Siblings were identified using KING software,^40^ with sibling-pairs identified based on the following criteria: kinship coefficient of between 0.177 and 0.355, the proportion of the genomes that share two alleles identical by descent (IBD) > 0.08 and the proportion of the genome that share zero alleles IBD > 0.04. Sibships of two or more siblings were constructed based on the identified sibling-pairs.

Educational attainment was measured using the following question: ‘What is your highest level of education?’ Participants answered one of five categories: (i) primary school, (ii) high school for 1 or 2 years, (iii) complete high school, (iv) college or university for <4 years and (v) college or university for ≥4 years. Participants with university degrees were assigned to 16 years of education, those who completed high school were assigned to 13 years, those who attended high school for 1 or 2 years were assigned to 12 years and those who only attended primary school were assigned to 10 years. Information on health outcome phenotypes (BMI, smoking, SBP, mortality) and genotyping for HUNT study participants is contained in the Supplementary methods (available as Supplementary data at *IJE* online).

### Finnish twin cohort

#### Overview

The older part of the Finnish twin cohort was established in 1974 by identifying pairs of persons born on the same day, in the same local community, of the same sex and with the same surname at birth from the population registers of Finland. The selection was restricted to twin pairs born before 1958 and the baseline analysis cohort consists of 16 282 pairs (32 564 twins). A baseline questionnaire was mailed in the autumn of 1975 with some data collection in early 1976. It contained questions relating to the assignment of zygosity as well as questions on various phenotypes including smoking behaviour, weight and height.^41^ All twins in the cohort were asked to participate in a second survey in 1981.

Data on educational attainment were collected in both the 1975 and 1981 questionnaires using the following questions: ‘What kind of education have you had, and what courses have you taken?’ The 1975 information was updated by the 1981 response if additional educational attainment was reported. Eight response categories ranging from less than primary school (4 years) to university education (17 years) provided by study participants were converted into years of education. The ninth response alternative was ‘Other’ and coded as missing (*n* = 587, 2.1% of participants). Years of education were then standardized to a mean of 0 and standard deviation of 1. Further information on Finnish twin cohort phenotypes is contained in the Supplementary methods (available as Supplementary data at *IJE* online).

A validated algorithm classified respondent pairs as monozygotic (MZ), dizygotic (DZ) or of unknown zygosity (XZ) (excluded from all analyses).^42^ Data on educational attainment and mortality were available for 27 229 individual twins living in Finland, which included 2779 individual twins (co-twin did not reply), 989 pairs of uncertain zygosity, 3518 MZ pairs and 7718 DZ pairs.

### Statistical analysis

#### Population and within-sibship models

The population model is a standard regression model in which the outcome is regressed (e.g. linear) on the exposure (educational attainment or educational attainment polygenic score: PGS). The within-sibship model is an extension to the population model including the mean sibship exposure value in the model, e.g. mean educational attainment value of each sibship. Each sibling’s exposure value is centred on the mean sibship exposure value. To account for relatedness between siblings, standard errors are clustered by sibship in both models using a sandwich estimator. More information on these models is contained in previous publications.^18^^,^^19^

Using individual-level data on 72 932 individuals from 32 351 sibships of European ancestry from UK Biobank (*n* = 40 734) and HUNT (*n* = 32 198) we estimated the association between measured educational attainment and outcomes (BMI, pack years of smoking, SBP, mortality) using population and within-sibship models. In population models, the outcome was regressed on educational attainment including relevant covariates. In within-sibship models, the mean educational attainment of each sibship was included as a covariate to account for variation in educational attainment between sibships. The continuous outcomes were chosen because they have previously been shown to be associated with educational attainment and because they were measured in the majority of UK Biobank and HUNT study participants. Linear regression models were used for BMI, pack years and SBP. Cox-proportional hazards models were used for mortality using date of birth as the baseline in UK Biobank and HUNT. Educational attainment, BMI, pack years and SBP were standardized after residualizing on birth year and sex.

We performed population and within-sibship PGS analyses using the UK Biobank and HUNT sibship data. The educational attainment PGS was constructed using weightings and directions of effect of independent variants identified at genome-wide significance (*P* < 5 × 10^–8^) in a BOLT-LMM^43^ GWAS of educational attainment in UK Biobank with the siblings excluded, as in a previous publication.^18^ The summary data were linkage disequilibrium clumped (*r*^2^ < 0.001, physical distance threshold = 10 000 kb, *P* < 5 × 10^–8^) in PLINK^44^ to generate 350 independent genetic variants. We regressed the resulting PGS on age and sex, and the standardized residuals (mean 0, SD = 1) were used in the analysis. In the population model, the outcome was regressed on the PGS. In the within-sibship model, the mean sibship PGS was included as a covariate to account for variation in parental genotypes. The PGS approach is equivalent to an inverse-variance weighted estimator from a summary-based two-sample MR analysis.^45^

#### UK Biobank and HUNT meta-analyses

Population and within-sibship models were fitted separately in UK Biobank and HUNT, and the estimates were meta-analysed using a fixed-effects model in the metafor R package. Shrinkage in estimates from the population to the within-sibship model was estimated as follows, with standard errors estimated using the delta method:


$$\frac{\beta^{\mathrm{Pop}}-\beta^{WS}}{\beta^{\mathrm{Pop}}}$$

#### UK Biobank and HUNT MR

MR estimates of the effects of educational attainment on the outcomes (BMI, pack years, SBP, mortality) were derived from the meta-analysis PGS association estimates (i.e. PGS–educational attainment, PGS–outcome). The point estimate was calculated using the Wald ratio of the PGS–outcome and PGS–educational attainment associations. Wald ratio standard errors were estimated using the delta method.

MR analyses require three core assumptions. First, genetic variants are strongly associated with the exposure (relevance); second, no unmeasured confounders of the association between the genetic variants and the outcome (independence); and third, genetic variants only influence the outcome via the exposure (exclusion–restriction).^46–48^ As discussed in previous work,^17^ MR estimates of categorical exposures such as educational attainment should generally be interpreted in terms of liability (e.g. liability to educational attainment) rather than effects of the categorical phenotype (e.g. years of schooling).

#### Within-sibship meta-analysis GWAS MR

We also performed two-sample MR analyses using within-sibship GWAS summary data from a recent within-sibship meta-analysis GWAS of 25 phenotypes.^18^ This study included data from UK Biobank, HUNT and an additional 16 cohorts (each with between *N* = 618 and 13 856). GWAS data were available for educational attainment (*N* = 128 777) as well as BMI (*N* = 140 883), SBP (*N* = 109 588), ever smoking (*N* = 124 791) and cigarettes per day in ever smokers (*N* = 28 134). These GWASs were conducted in the same studies so there is near-complete sample overlap between the different GWASs.

As genetic instruments, we used the same 350 genetic variants as in the UK Biobank and HUNT analyses described above, which were derived from a BOLT-LMM GWAS of educational attainment in UK Biobank with the siblings excluded (*P* < 5 × 10^–8^, *r*^2^ < 0.001, physical distance threshold = 10 000 kb). Using the within-sibship meta-analysis GWAS data we then derived MR effect estimates ($\beta_{MR}$) of educational attainment on the four health outcomes in both population and within-sibship models using an inverse-variance weighted approach^18^ as follows:


$$\beta_{MR}=\sum_{1}^{n}\frac{\beta_{EA}^{k}\;*\;\beta_{\mathrm{OUT}}^{k}}{(\sigma_{\mathrm{Out}}^{k}{)}^{2}}/\sum_{1}^{n}\frac{{(\beta }_{EA}^{k}{)}^{2}}{(\sigma_{\mathrm{Out}}^{k}{)}^{2}}$$

where $\beta_{EA}$ represents the association estimate from educational attainment GWAS, $\beta_{\mathrm{Out}}$ represents the association estimate from outcome GWAS, $\sigma_{\mathrm{Out}}$ represents the standard error from outcome GWAS, n represents the number of genetic variants and k represents the *k*-th variant.

The standard error of $\beta_{MR}$ was estimated as follows:


$$SE\left(\beta_{MR}\right)=\sqrt{\frac{1}{\sum_{1}^{n}\frac{{(\beta }_{EA}^{k}{)}^{2}}{(\sigma_{\mathrm{Out}}^{k}{)}^{2}}}}$$

where n represents the number of genetic variants and k represents the *k*-th variant.

#### Finnish twin cohort analysis

UK Biobank and HUNT analyses included both twin and non-twin siblings, with the vast majority (>95%) being non-twin siblings of different ages—a potential concern with educational attainment trends changing over time. We investigated whether the observed inverse association between educational attainment and mortality persisted in twin-only analyses using data on 27 229 individuals from the Finnish twin cohort that included 3518 MZ and 7718 DZ twin pairs.

The association between educational attainment and mortality was estimated in the whole sample (*N* = 27 229) using Cox-proportional hazards models with adjustment for sex and smoking (population model). Stratified Cox-proportional hazard models were applied to MZ and DZ twins separately with baseline hazards stratified by twin pair, with adjustment for smoking. All twin pairs included were of the same sex. All analyses were performed in Stata using the stcox package.

## Results

### Phenotypic educational attainment, health outcomes and mortality in UK Biobank and HUNT

**Figure 2:**
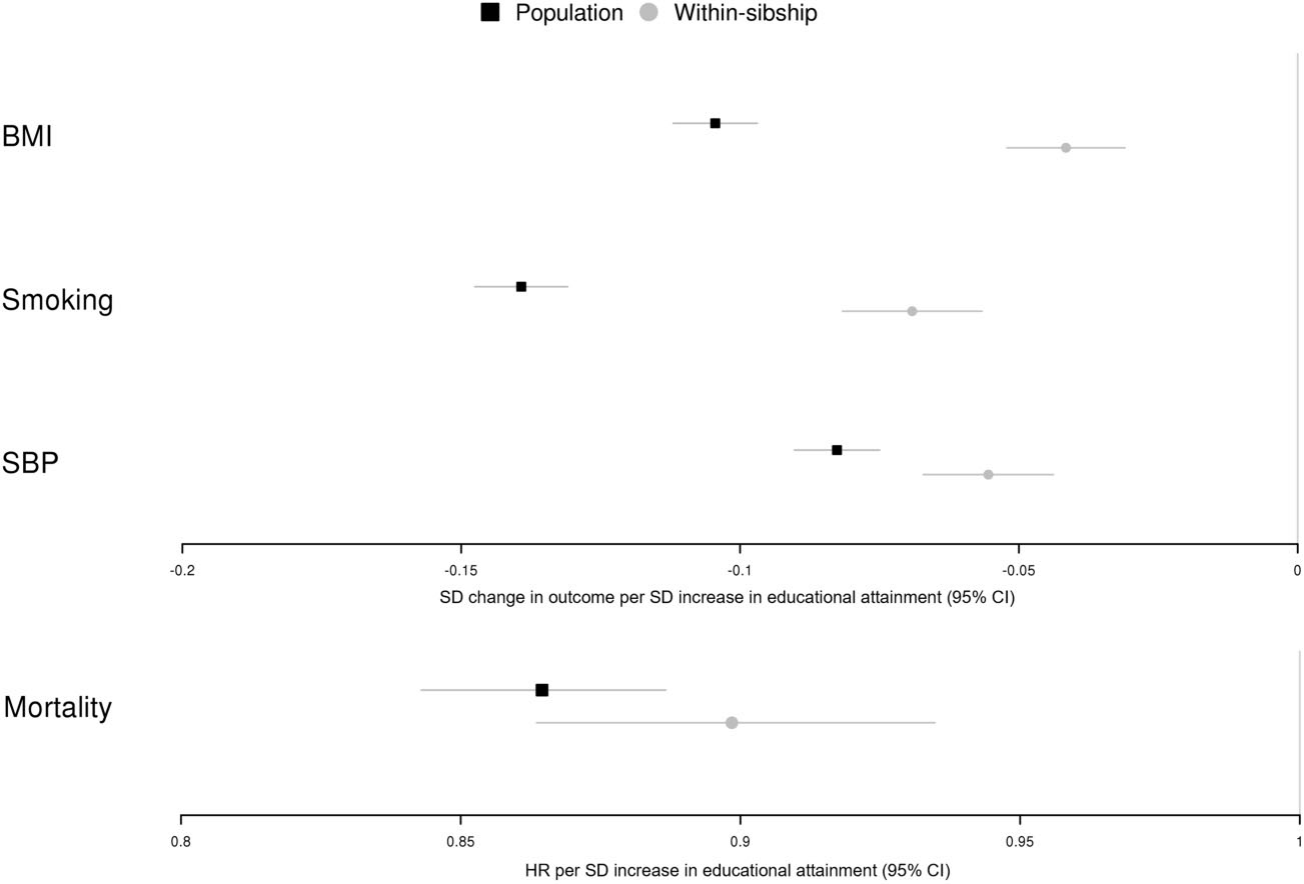
Phenotypic educational attainment and health outcomes. Figure 2 shows associations between phenotypic educational attainment (years in full-time education derived using qualifications) and body mass index, smoking (cigarettes, measured in pack years), systolic blood pressure and mortality in the population and within-sibship models in UK Biobank and HUNT. Estimates for mortality are presented as hazard ratios with the rest of the estimates presented in standard deviation units. BMI, body mass index; SBP, systolic blood pressure

Higher educational attainment was strongly associated with lower BMI, pack years of smoking, SBP and mortality in both population and within-sibship models. In within-sibship models, a 1-SD higher educational attainment (corresponding to an additional 2.3 years in UK Biobank and 1.2 years in HUNT) was associated with lower BMI (0.04 SD; 95% CI 0.03, 0.05), fewer pack years of cigarette smoking (0.10 SD; 95% CI 0.08, 0.12), lower SBP (0.06 SD; 95% CI 0.04, 0.07) and lower mortality [hazard ratio (HR) 0.90; 95% CI 0.86, 0.93]. Population estimates that did not account for family-level confounding were in the same direction but substantially larger (34% for mortality to 146% for BMI) than the within-sibship point estimates (Figure 2 and Supplementary Table S1, available as Supplementary data at *IJE* online).

### Phenotypic educational attainment and mortality in the Finnish twin cohort

In non-twin population regression models, using data from the whole sample, 1-SD higher educational attainment was strongly associated with lower mortality (HR 0.95; 95% CI 0.93, 0.97) after adjusting for sex and smoking. Estimates from the within DZ twin pair (HR 0.91; 95% CI 0.83, 1.01) and within MZ twin pair (HR 0.87; 95% CI 0.70, 1.08) analyses were broadly consistent with the Finnish twin cohort population estimate as well as the UK Biobank and HUNT within-sibship estimate (HR 0.90; 95% CI 0.86, 0.93) but confidence intervals overlap with the null hypothesis. In sex-stratified twin analyses, point estimates were larger in magnitude for males than for females (Supplementary Table S2, available as Supplementary data at *IJE* online).

### Educational attainment PGS, educational attainment, health outcomes and mortality in UK Biobank and HUNT

**Figure 3:**
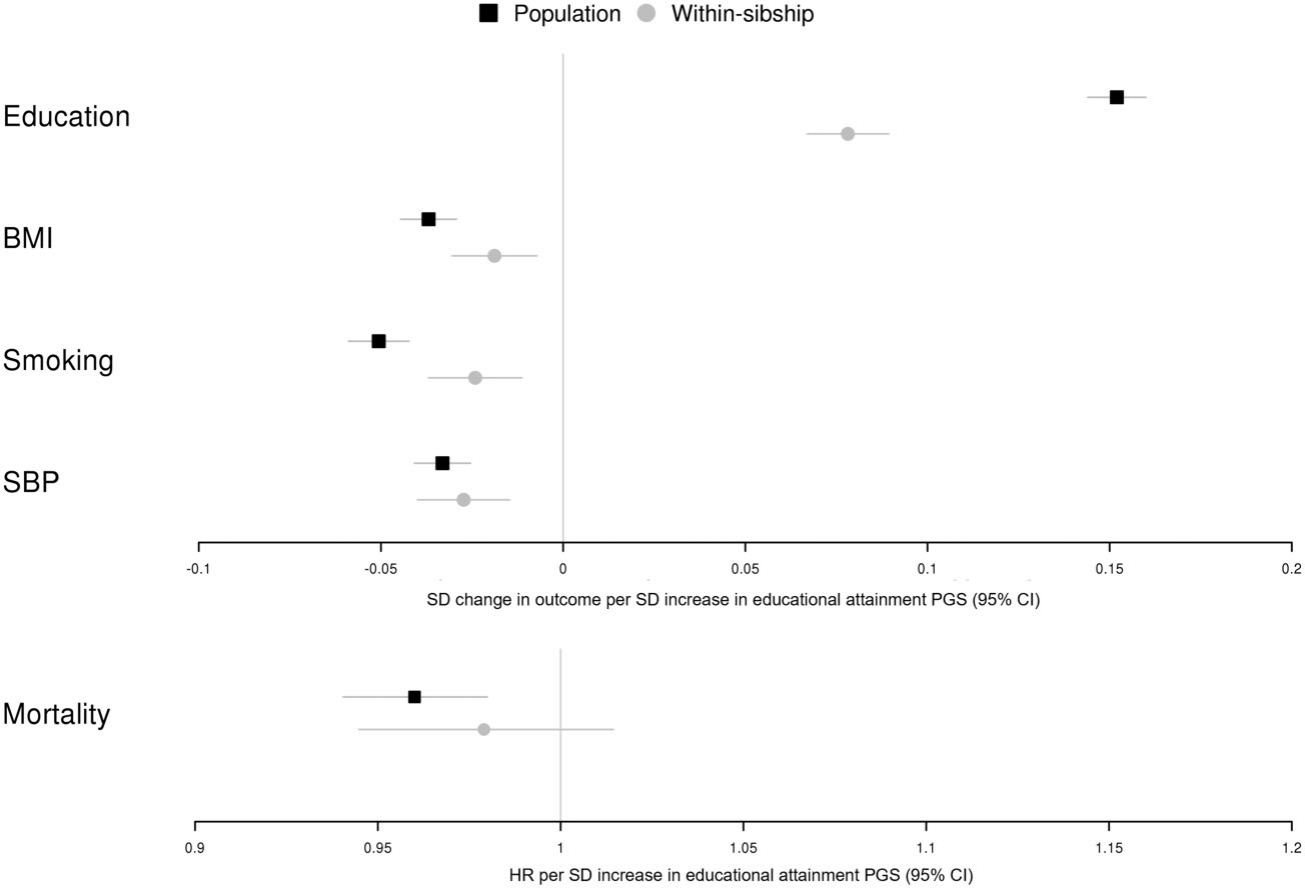
Educational attainment polygenic score (PGS), educational attainment and health outcomes. Figure 3 shows associations between an educational attainment PGS and education (measured educational attainment), body mass index, cigarette smoking (pack years), systolic blood pressure and mortality in the population and within-sibship models in UK Biobank and HUNT. Estimates for mortality are presented as hazard ratios per standard deviation increase in the polygenic score with the rest of the outcome estimates presented in standard deviation units

The educational attainment PGS was strongly associated with educational attainment in both population and within-sibship models. Consistently with previous studies,^27^^,^^29^^,^^49^ the population PGS association estimate attenuated by 49% (95% CI 40%, 58%) in the within-sibship model. In the population model, a higher educational attainment PGS was associated with lower BMI, fewer pack years of cigarette smoking, lower SBP and lower mortality. In the within-sibship model, the PGS was associated with BMI, cigarette smoking and SBP in the same direction but there was limited evidence for an association with mortality, likely because of lower statistical power. The within-sibship PGS association estimates for BMI and cigarette smoking were 49% (95% CI 16%, 82%) and 52% (95% CI 26%, 79%) smaller than the population PGS estimates, respectively, consistently with the within-sibship attenuations for educational attainment (Figure 3 and Supplementary Tables S3 and S4, available as Supplementary data at *IJE* online).

### MR of educational attainment on health outcomes and mortality using UK Biobank and HUNT PGS estimates

**Figure 4:**
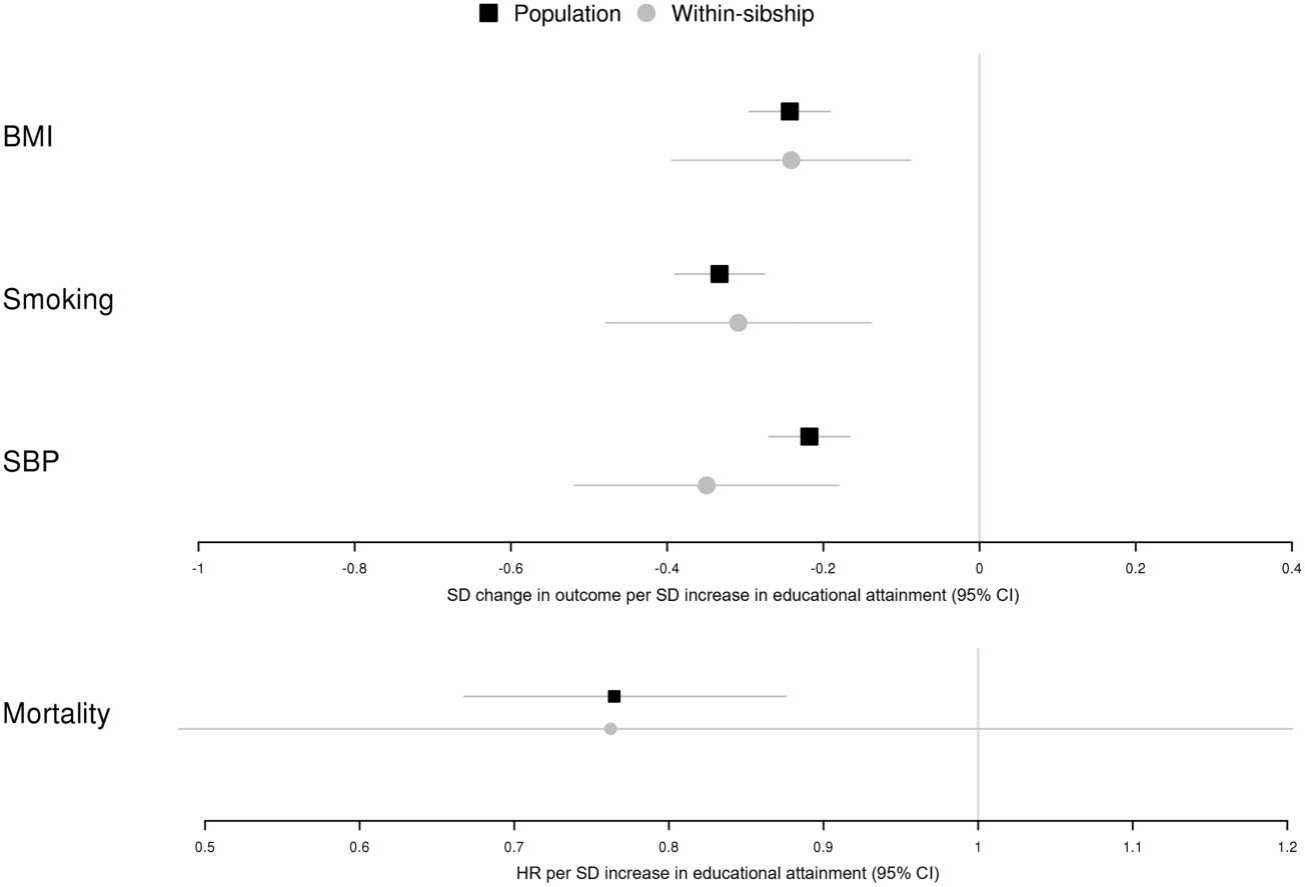
Mendelian randomization (MR) estimates of educational attainment on health outcomes from UK Biobank and HUNT. Figure 4 shows population and within-sibship MR estimates of the effect of educational attainment on body mass index, smoking (pack years of cigarette smoking), systolic blood pressure and mortality. These estimates were derived using the polygenic score association estimates in Figure 3 from the UK Biobank and HUNT studies. Estimates are presented in standard deviation units for body mass index, smoking and systolic blood pressure, and as hazard ratios for mortality

Population MR estimates indicated that a 1-SD increase in liability to educational attainment reduced BMI by 0.24 SD units (95% CI 0.19, 0.29), pack years of cigarette smoking by 0.33 SD (95% CI 0.28, 0.39) and SBP by 0.22 SD (95% CI 0.17, 0.27) (Figure 4). Within-sibship MR estimates were consistent with the population MR estimates for BMI (0.24; 95% CI 0.09, 0.39), cigarette smoking (0.31; 95% CI 0.14, 0.48) and SBP (0.35; 95% CI 0.18, 0.52). The population and within-sibship MR point estimates for mortality were also consistent (HR per SD increase in educational attainment; population 0.76; 95% CI 0.67, 0.88; within-sibship 0.76; 95% CI 0.48, 1.20) but the imprecision of the within-sibship estimate prevented stronger conclusions.

Differences between population and within-sibship MR estimates are a function of differences in the PGS–outcome and PGS–educational attainment estimates. If the PGS estimates change by the same proportion from the population model to the within-sibship model, then the population and within-sibship MR estimates will be consistent (Figure 4 and Supplementary Tables S4 and S5, available as Supplementary data at *IJE* online).

### MR of educational attainment on health outcomes using within-sibship GWAS summary data

**Figure 5:**
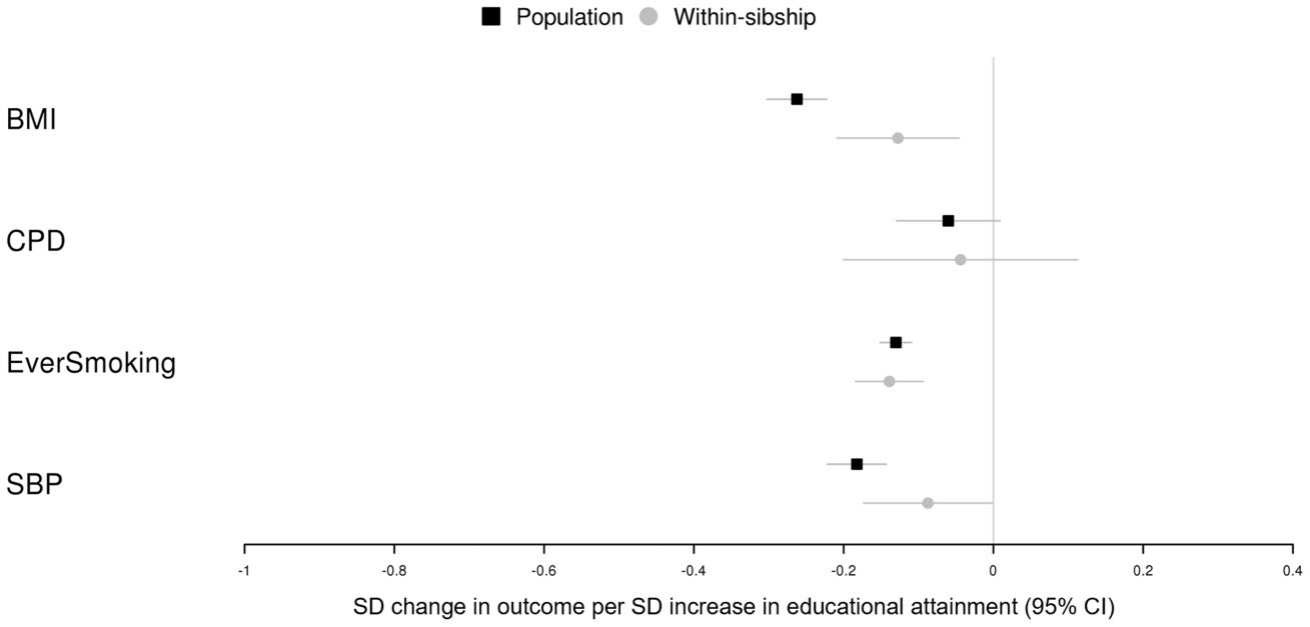
Mendelian randomization estimates of educational attainment on health outcomes using summary data from the within-sibship Genome-wide Association Study (GWAS). Figure 5 shows population and within-sibship Mendelian randomization estimates (inverse-variance weighted) of the effect of educational attainment on body mass index, cigarettes per day measured in ever smokers only, ever smoking and systolic blood pressure. These estimates were derived using GWAS summary statistics from a within-sibship meta-analysis GWAS of ≤18 studies. Estimates are presented in standard deviation units except for ever smoking (binary) where estimates are in terms of risk difference %. BMI, body mass index; CPD, cigarettes per day; SBP, systolic blood pressure

Population and within-sibship MR estimates based on the within-sibship GWAS summary data provided further evidence that higher educational attainment lowers BMI, risk of ever smoking and SBP. Confidence intervals for cigarettes per day overlapped the null in both models but statistical power was limited because data were only collected in ever smokers. There was some evidence that the within-sibship MR estimates were smaller than the population estimates for BMI (51%; 95% CI 19%, 83%) and SBP (52%; 95% CI 4%, 100%), which was not observed in the UK Biobank and HUNT analyses. The standard errors for the within-sibship MR estimates for BMI and SBP were 47% and 49% smaller, respectively, in the two-sample MR analyses compared with the UK Biobank and HUNT analyses because of the larger sample size of the within-sibship GWAS (Figure 5 and Supplementary Table S6, available as Supplementary data at *IJE* online).

## Discussion

We used within-sibship MR to provide evidence that higher liability to educational attainment reduces BMI, cigarette smoking and SBP. These findings strengthen evidence for beneficial effects of educational attainment, or closely correlated trait, on adulthood health by illustrating that previously observed effects persist when population stratification, assortative mating and indirect genetic effects from parents are controlled for in within-sibship MR.

An important consideration for MR analyses of educational attainment is that genetic variants instrument liability to educational attainment, a latent measure of educational attainment, rather than specific measured educational attainment phenotypes (e.g. having a university degree)^17^^,^^50^ or other related traits (e.g. cognition). Genetic variants influence measured education phenotypes via their effect on liability to educational attainment and so genetic association estimates will capture all effects of liability, which may or may not act via changes in the measured education phenotype. For example, an educational-attainment-increasing genetic variant may influence an outcome because the variant increases the probability that an individual attains a measured qualification such as a university degree, but also because the variant influences related unmeasured characteristics such as choice of educational track, a personality phenotype or cognitive ability.

The distinction between measured education phenotypes and liability to educational attainment is particularly relevant in the within-sibship model because siblings often have genotype differences for educational attainment variants but often have the same measured educational attainment values (e.g. both siblings attended university). A conventional MR analysis assumes that genetic differences between siblings do not affect the outcome if the siblings have the same value for the measured education phenotype. This is implausible as genetic variants are likely to influence unmeasured differences between siblings such as the choice of degree. Therefore, we interpret the within-sibship MR results as providing evidence that an underlying liability to education has beneficial effects on health, rather than specific educational attainment qualifications.^17^^,^^51^

Consistently with previous studies,^18^^,^^26^^,^^52^ the association estimate between the educational attainment PGS and educational attainment attenuated on average by around a half from the population model to the within-sibship model when using weights from a GWAS of unrelated individuals. However, the association estimates of educational attainment PGS on health outcomes were also attenuated by a similar degree. As the attenuation was balanced, the population and within-sibship MR effect estimates, which are a ratio of single nucleotide polymorphism (SNP) (or PGS)–outcome and SNP–exposure associations, were consistent. These results illustrate how population stratification, assortative mating and indirect genetic effects can distort genetic association estimates but will not necessarily affect MR estimates if the gene–exposure and gene–outcome association estimates are affected proportionally.

A caveat of our work is that MR estimates are sensitive to the assumption that genetic variants only influence the outcome via their effect on (liability to) educational attainment (exclusion–restriction), which could be violated by directional pleiotropy. Previous work has illustrated that MR with measured educational attainment is unlikely to satisfy this assumption, despite use of pleiotropy-robust methods.^16^ We presented estimates in terms of liability to educational attainment to acknowledge the likelihood of such effects but note that, in the context of interventions, effects of liability to educational attainment are less useful than effects of specific education phenotypes. Future studies could use multivariable MR and structural equation modelling approaches to disentangle mechanisms underlying our results by exploring potential pleiotropic pathways potentially relating to both cognitive and non-cognitive phenotypes.^53^^,^^54^

Our work has further limitations. First, our within-sibship MR estimate for mortality was imprecise because mortality data were only available in UK Biobank and HUNT. Second, educational attainment is known to influence participation in biobanks so our study may have been susceptible to selection bias.^55^ Third, the gene–exposure and gene–outcome estimates in the within-sibship GWAS two-sample MR analyses were from largely overlapping samples, which could have potentially induced modest bias.^56^ Fourth, there is evidence that within-sibship models using PGS based on weights from population GWAS could introduce bias.^57^ This could have potentially affected our MR estimates from the individual-level PGS approach in UK Biobank and HUNT but not our MR estimates using the within-sibship meta-analysis GWAS data where the gene–exposure and gene–outcome estimates were derived from within-sibship models. Estimates from the two different MR approaches provided consistent qualitative evidence of effects of educational attainment on the tested outcomes suggesting that this potential limitation is unlikely to have affected our overall conclusions. There were some quantitative differences between the two approaches, with evidence of within-sibship shrinkage from the summary-based MR estimates of educational attainment on BMI and SBP but not from the PGS approach. Fifth, within-sibship models do not control for indirect genetic effects of siblings. Previous studies have indicated that sibling indirect genetic effects are likely to be small, suggesting that they are unlikely to have impacted our findings.

We found compelling evidence that educational attainment (or liability to educational attainment) influences BMI, smoking and SBP, even after accounting for population stratification, assortative mating and indirect genetic effects of parents. Within-family MR more closely emulates a randomized experiment because of random variation in meiotic segregation within families^31^ but has been historically limited by data availability. The emerging availability of within-family GWAS data will enable researchers to better disentangle the effects of social and behavioural phenotypes on health outcomes.

## Ethics approval

This research has been conducted using the UK Biobank resource under Application Number 8786. UK Biobank obtained ethics approval from the North West Multi-centre Research Ethics Committee and obtained informed consent from all study participants. The use of HUNT data in this study was approved by the Regional Committee for Ethics in Medical Research, Central Norway (2017/2479). All participants signed informed consent for participation and the use of data in research. Register linkages and use of the questionnaire data were approved by the Ethics Committee at the Finnish Institute of Health and Welfare (THL 220/6.02.04/2021).

## Supplementary Material

dyad079_Supplementary_Data

## Data Availability

UK Biobank individual-level participant data are available via enquiry to access@ukbiobank.ac.uk. Researchers associated with Norwegian research institutes can apply for the use of HUNT data and samples with approval by the Regional Committee for Medical and Health Research Ethics. Researchers from other countries may apply if collaborating with a Norwegian Principal Investigator. Information for data access can be found at https://www.ntnu.edu/hunt/data. The HUNT variables are available for browsing on the HUNT databank at https://hunt-db.medisin.ntnu.no/hunt-db/. Use of the full genetic data set requires the use of an approved secure computing solution such as the HUNT Cloud (https://docs.hdc.ntnu.no/). Example scripts for population and within-sibship models are available on GitHub https://github.com/LaurenceHowe/EducationSiblingMR/. Summary data from the within-sibship meta-analysis GWAS are publicly available for download on OpenGWAS (https://gwas.mrcieu.ac.uk/) via the TwoSampleMR R package. Note that the summary data include both ‘population’ and ‘within-sibship’ estimates for each phenotype, with the model detailed in the metadata notes.
