# Supplementary material for: Educational attainment, health outcomes and mortality: a within-sibship Mendelian randomization study
Source: Int J Epidemiol. 2023 Jun 9;52(5):1579–91. doi: 10.1093/ije/dyad079 (PMC10555788; doi:10.1093/ije/dyad079)
Supplement: dyad079_Supplementary_Data [file dyad079_supplementary_data.docx]

## Members of the Within-Family Consortium (WFC)

Rafael Ahlskog, Ole A Andreassen, Helga Ask, Archie Campbell, Rosa Cheesman, Yoonsu Cho, Kaare Christensen, Elizabeth C Corfield, Christina C Dahm, Alexandra Havdahl, William D Hill, Shona M Kerr, Antti Latvala, Marianne Nygaard, Teemu Palviainen, Nancy L Pedersen, Robert Plomin, Melissa C Southey, Camilla Stoltenberg.

**Supplementary Tables**

**Table S1** Measured educational attainment (years of full-time education), health outcomes and mortality

| **Outcome**  **(units)** | **Model** | **Change in outcome per SD increase in measured educational attainment (95% CI)** | | |
| --- | --- | --- | --- | --- |
|  |  | **UK Biobank**  (n = 40,734) | **HUNT**  (n = 32,198) | **Meta-analysis** |
| BMI (SD) | Population | -0.14 (-0.15, -0.13) | -0.07 (-0.08, -0.05) | -0.11 (-0.11, -0.10) |
|  | Within-sibship | -0.04 (-0.06, -0.03) | -0.04 (-0.06, -0.03) | -0.04 (-0.05, -0.03) |
| Pack years of smoking (SD) | Population | -0.16 (-0.17, -0.15) | -0.11 (-0.12, -0.10) | -0.14 (-0.15, -0.13) |
|  | Within-sibship | -0.10 (-0.12, -0.08) | -0.04 (-0.06, -0.02) | -0.07 (-0.08, -0.06) |
| SBP (SD) | Population | -0.07 (-0.08, -0.06) | -0.10 (-0.11, -0.09) | -0.08 (-0.09, -0.07) |
|  | Within-sibship | -0.04 (-0.06, -0.02) | -0.07 (-0.09, -0.05) | -0.06 (-0.07, -0.04) |
| Mortality (HR) | Population | 0.83 (0.80, 0.87) | 0.89 (0.86, 0.91) | 0.87 (0.84, 0.89) |
|  | Within-sibship | 0.88 (0.81, 0.95) | 0.91 (0.86, 0.95) | 0.90 (0.86, 0.93) |

^BMI = body mass index, SD = standard deviation, SBP = systolic blood pressure, HR = hazard ratio^

**Table S2** Educational attainment (years of full-time education) and mortality in the Finnish Twin Cohort

| **Model** | **HR per SD increase in measured educational attainment (95% CI)** | | |
| --- | --- | --- | --- |
|  | **Men** | **Women** | **Men and Women** |
| Population model:  HR (95% CI)  n = 27,229 individuals | 0.94 (0.91, 0.96) | 0.97 (0.94, 1.01) | 0.95 (0.93, 0.97) |
| DZ twins:  HR (95% CI)  n = 7,718 pairs | 0.96 (0.84, 1.09) | 0.84 (0.72, 0.99) | 0.91 (0.83, 1.01) |
| MZ twins:  HR (95% CI)  n = 3,518 pairs | 0.95 (0.72, 1.26) | 0.78 (0.56, 1.07) | 0.87 (0.70, 1.08) |

^HR = hazard ratio, DZ = dizygotic, MZ = monozygotic^

**Table S3** Educational attainment polygenic score, educational attainment (years of full-time education), health outcomes and mortality

| **Outcome (units)** | **Model** | **Change in outcome per SD increase in educational attainment PGS (95% CI)** | | |
| --- | --- | --- | --- | --- |
|  |  | **UK Biobank**  (n = 40,734) | **HUNT**  (n = 32,198) | **Meta-analysis** |
| Educational attainment (SD) | Population | 0.17 (0.16, 0.18) | 0.13 (0.11, 0.14) | 0.15 (0.14, 0.16) |
|  | Within-sibship | 0.07 (0.06, 0.09) | 0.08 (0.07, 0.10) | 0.08 (0.07, 0.09) |
| BMI (SD) | Population | -0.05 (-0.06, -0.04) | -0.02 (-0.03, -0.00) | -0.04 (-0.04, -0.03) |
|  | Within-sibship | -0.02 (-0.04, -0.01) | -0.01 (-0.03, 0.00) | -0.02 (-0.03, -0.01) |
| Pack years of smoking (SD) | Population | -0.05 (-0.06, -0.04) | -0.06 (-0.07, -0.04) | -0.05 (-0.06, -0.04) |
|  | Within-sibship | -0.02 (-0.04, -0.00) | -0.02 (-0.04, -0.01) | -0.02 (-0.04, -0.01) |
| SBP (SD) | Population | -0.03 (-0.04, -0.02) | -0.04 (-0.05, -0.02) | -0.03 (-0.04, -0.03) |
|  | Within-sibship | -0.03 (-0.04, -0.01) | -0.03 (-0.05, -0.01) | -0.03 (-0.04, -0.01) |
| Mortality (HR) | Population | 1.00 (0.95, 1.04) | 0.95 (0.91, 0.99) | 0.96 (0.94, 0.98) |
|  | Within-sibship | 0.99 (0.91, 1.07) | 0.98 (0.94, 1.02) | 0.98 (0.94, 1.01) |

^PGS = polygenic score, SD = standard deviation, BMI = body mass index, SBP = systolic blood pressure, HR = hazard ratio^

**Table S4** Within-sibship attenuations in associations between educational attainment PGS and outcomes.

| **Outcome (units)** | **Attenuation (95% CI)** |
| --- | --- |
| Educational attainment (SD) | 49% (41%, 56%) |
| BMI (SD) | 49% (16%, 82%) |
| Pack years of smoking (SD) | 52% (26%, 79%) |
| SBP (SD) | 18% (-25%, 60%) |
| Mortality (HR) | 48% (-43%, 139%) |

^SD = standard deviation, BMI = body mass index, SBP = systolic blood pressure, HR = hazard ratio^

**Table S5** Mendelian randomization estimates of educational attainment (years of full-time education) on health outcomes and mortality from UK Biobank and HUNT

| **Outcome**  **(units)** | **Change in outcome per SD increase in educational attainment** | |
| --- | --- | --- |
|  | **Population estimate:**  **(95% CI)** | **Within-sibship estimate:**  **(95% CI)** |
| BMI (SD) | -0.24 (-0.29, -0.19) | -0.24 (-0.40, -0.09) |
| Pack years of smoking (SD) | -0.33 (-0.39, -0.28) | -0.31 (-0.48, -0.14) |
| SBP (SD) | -0.22 (-0.27, -0.17) | -0.35 (-0.52, -0.18) |
| Mortality (HR) | 0.76 (0.67, 0.88) | 0.76 (0.48, 1.20) |

^SD = standard deviation, BMI = body mass index, SBP = systolic blood pressure, HR = hazard ratio^

**Table S6** Mendelian randomization estimates of educational attainment (years of full-time education) on health outcomes from the within-sibship meta-analysis GWAS

| **Outcome**  **(units)** | **Change in outcome per SD increase in educational attainment** | |
| --- | --- | --- |
|  | **Population estimate:**  **(95% CI)** | **Within-sibship estimate:**  **(95% CI)** |
| BMI (SD) | -0.26 (-0.30, -0.22) | -0.13 (-0.21, -0.05) |
| CPD (SD) | -0.06 (-0.13, 0.01) | -0.04 (-0.20, 0.11) |
| Ever smoking (risk increase) | -0.13 (-0.15, -0.11) | -0.14 (-0.18, -0.09) |
| SBP (SD) | -0.18 (-0.22, -0.14) | -0.09 (-0.17, -0.00) |

^GWAS = genome-wide association study, SD = standard deviation, BMI = body mass index, CPD = cigarettes per day, SBP = systolic blood pressure^

## Supplementary Methods

### UK Biobank siblings

The UK Biobank study sample incidentally includes many related individuals. In our analyses we included individuals with one or more full siblings in the study sample. Siblings were identified in a previous study using the UK Biobank derived estimates of pairwise identical by state (IBS) kinships and the proportion of unshared loci (IBS0) (1). Briefly, to identify sibling pairs, we considered first that sibling pairs should have IBS0 > 0, unlike parent-offspring pairs, and should have an expected IBS kinship of 0.5 with a standard deviation of 0.038, useful to distinguish sibling pairs from more distant half siblings as well as avuncular and cousin pairs. Plotting IBS0 against IBS we visually identified a cluster of pairwise relationships that satisfied these criteria and labelled the individuals as sibling pairs if they fell within the following bounds (IBS: > 0.5-21*IBS0, < 0.7) and (IBS0: >0.001, <0.008) (2). After restricting the sample to sibships with two or more individuals with educational attainment data, our analysis sample included 40,734 individuals from 19,773 sibships.

### UK Biobank phenotypes

Educational attainment was defined as in a previous study (3), using the self-reported qualifications from questionnaire data (field ID: 6138-0.0) to estimate the number of years each individual spent in full-time education. For example, “College or University degree” was mapped to 17 years while “A levels/AS levels or equivalent” was mapped to 14 years. Where individuals reported multiple qualifications, the highest qualification in terms of years in education was used. BMI was derived from measures of standing height and weight (field ID: 21001.0.0). Pack years of smoking (field ID 20161-0.0) was derived using smoking intensity and behaviour data from questionnaire data. SBP was measured using an automated reading from an Omron Digital blood pressure monitor (field ID: 4080-0.0). Mortality data (date of death, field ID: 40000-0.0) was obtained via linkage with the UK death registry with a median follow-up of 10.0 years from study enrolment with 4.0% of the cohort experiencing a fatal event during follow-up.

### UK Biobank genotyping

UK Biobank study participants (N= 488,377) were genotyped using the UK BiLEVE (N= 49,950) and the closely related UK Biobank Axiom™ Arrays (N= 438,427). Directly genotyped variants were pre-phased using SHAPEIT3 (4) and imputed using Impute4 and the UK10K (5), Haplotype Reference Consortium (6) and 1000 Genomes Phase 3 (7) reference panels (8, 9).

### HUNT phenotypes

Participants’ height and weight (used to compute BMI) were measured with participants wearing light clothes without shoes to the nearest centimetre and half kilogram, respectively. Educational attainment was measured using the following question ‘What is your highest level of education?’. Participants answered one of five categories (1) primary school, (2) high school for 1 or 2 years, (3) complete high school, (4) college or university less than 4 years, and (5) college or university 4 years or more. Participants with university degrees were assigned to 16 years of education, those who completed high school were assigned 13 years, those who attended high school for 1 or 2 years were assigned to 12 years, and those who only attended primary school were assigned to 10 years. Smoking intensity as packs per year was derived from the smoking habit questionnaire data. Here pack-years as a cumulative measure of smoking exposure were calculated using number of cigarettes smoked daily multiplied by the years of smoking divided by 20 (number of cigarettes in a pack).

SBP was measured using automated oscillometry (Critikon Dinamap 845XT and XL9301, acquired by GE Medical Systems Information Technologies in 2000) on the right arm in a relaxed sitting position (10, 11). SBP was measured twice with a one-minute interval between measurements with the mean of both measurements used in this study. For all phenotypes, if measured both in the HUNT2 and HUNT3 surveys, then the measurement from HUNT2 were preferred over the same measurement from HUNT3 because the HUNT2 survey had a larger sample size. Data on all-cause mortality was provided by the Norwegian National Registry with data available up until 15^th^ July 2020 and 28.9% (20,102 individuals) of HUNT2 and HUNT3 experiencing a fatal event during follow-up.

### HUNT genotyping

DNA available from 71,860 HUNT samples from HUNT2 and HUNT3 and were genotyped (10) using three Illumina HumanCoreExome arrays: HumanCoreExome12 v1.0 (n= 7570), HumanCoreExome12 v1.1 (n=4960) and University of Michigan HUNT Biobank v1.0 (n=58041; HumanCoreExome-24 v1.0, with custom content). Quality control was performed separately for genotype data from different arrays. The call rate of genotyped samples was >99%. Imputation was performed on samples of recent European ancestry using Minimac3 (v2.0.1, <http://genome.sph.umich.edu/wiki/Minimac3>) (12) from a merged reference panel constructed from i) the Haplotype Reference Consortium panel (release version 1.1)(6) and ii) a local reference panel based on 2,202 whole-genome sequenced HUNT participants(13). Subjects included in this study were of European ancestry and passed quality control.

### Finnish twin cohort phenotypes

Data on educational attainment were collected in both the 1975 and 1981 questionnaires using the following questions: “What kind of education have you had, and what courses have you taken?”. The 1975 information was updated by the 1981 response if additional educational attainment was reported. Eight response categories ranging from less than primary school (4 years) to university education (17 years) provided by study participants were converted into years of education. The ninth response alternative was *Other* and coded as missing (n=587, 2.1% of participants). Years of education were then standardized to a mean of zero and standard deviation of one.

Data on cigarette smoking history were obtained in 1975 and 1981 in an identical fashion. Smoking status was classified as never smoking (less than 100 cigarettes lifetime), occasional smoking (never regular daily or almost daily smoking), former smokers (regular smokers who did not smoke at the time of the survey) and current smokers, who smoked daily or almost daily. Daily smokers were further grouped as light (1-9 cigarettes per day (CPD), moderate (10-19 CPD) or heavy (20 or more CPD) smokers. Pack-years were computed from years smoked (current age or age at cessation minus age at initiation) and amount smoked divided by 20.

Mortality and vital status follow-up were based on record-linkage with the Population Information System database on all residents of Finland, and with the Cause-of-Death register, Statistics Finland. Mortality follow-up started May 1, 1976 and ended upon permanent emigration from Finland, death or end of follow-up on December 31, 2018.

**Supplementary References**

1. Brumpton B, Sanderson E, Hartwig FP, Harrison S, Vie GÅ, Cho Y, et al. Within-family studies for Mendelian randomization: avoiding dynastic, assortative mating, and population stratification biases. Nature Communications. 2020:602516.

2. Hill WG, Weir BS. Variation in actual relationship as a consequence of Mendelian sampling and linkage. Genetics research. 2011;93(1):47-64.

3. Okbay A, Beauchamp JP, Fontana MA, Lee JJ, Pers TH, Rietveld CA, et al. Genome-wide association study identifies 74 loci associated with educational attainment. Nature. 2016;533(7604):539.

4. O'Connell J, Sharp K, Shrine N, Wain L, Hall I, Tobin M, et al. Haplotype estimation for biobank-scale data sets. Nature Genetics. 2016;48(7):817-20.

5. UK10K Consortium. The UK10K project identifies rare variants in health and disease. Nature. 2015;526(7571):82-90.

6. McCarthy S, Das S, Kretzschmar W, Delaneau O, Wood AR, Teumer A, et al. A reference panel of 64,976 haplotypes for genotype imputation. Nature genetics. 2016;48(10):1279.

7. Genomes Project Consortium. A global reference for human genetic variation. Nature. 2015;526(7571):68-74.

8. Bycroft C, Freeman C, Petkova D, Band G, Elliott LT, Sharp K, et al. The UK Biobank resource with deep phenotyping and genomic data. Nature. 2018;562(7726):203.

9. Mitchell RE, Hemani G, Dudding T, Paternoster L. UK Biobank Genetic Data: MRC-IEU Quality Control, version 1, 13/11/2017 2017 [

10. Krokstad S, Langhammer A, Hveem K, Holmen T, Midthjell K, Stene T, et al. Cohort profile: the HUNT study, Norway. International journal of epidemiology. 2012;42(4):968-77.

11. Holmen J, Midthjell K, Krüger Ø, Langhammer A, Holmen TL, Bratberg GH, et al. The Nord-Trøndelag Health Study 1995–97 (HUNT 2): objectives, contents, methods and participation. Norsk epidemiologi. 2003;13(1):19-32.

12. Das S, Forer L, Schönherr S, Sidore C, Locke AE, Kwong A, et al. Next-generation genotype imputation service and methods. Nature Genetics. 2016;48(10):1284.

13. Zhou W, Fritsche LG, Das S, Zhang H, Nielsen JB, Holmen OL, et al. Improving power of association tests using multiple sets of imputed genotypes from distributed reference panels. Genetic epidemiology. 2017;41(8):744-55.
